# Ten-Year Outcomes in Patients Undergoing Simultaneous Coronary and Renal Angiography—Does Renal Artery Stenosis Matter?

**DOI:** 10.3390/jcm13123374

**Published:** 2024-06-07

**Authors:** Adam Kern, Tomasz Stompór, Krystian Bojko, Ewa Sienkiewicz, Sebastian Pawlak, Dariusz Pawlak, Grzegorz Poskrobko, Ewa Andrasz, Leszek Gromadziński, Rakesh Jalali, Dariusz Onichimowski, Grażyna Piwko, Artur Zalewski, Jacek Bil

**Affiliations:** 1 Department of Cardiology and Internal Medicine, School of Medicine, Collegium Medicum, University of Warmia and Mazury in Olsztyn, 10-727 Olsztyn, Poland; krystian.bojko@uwm.edu.pl (K.B.); sebastian.pawlak@uwm.edu.pl (S.P.); leszek.gromadzinski@uwm.edu.pl (L.G.); 2 Department of Cardiology, Regional Specialist Hospital in Olsztyn, 10-045 Olsztyn, Polandgposkrobko@gmail.com (G.P.); ewa8801@wp.pl (E.A.); 3 Department of Nephrology, Hypertension and Internal Medicine, School of Medicine, Collegium Medicum, University of Warmia and Mazury in Olsztyn, 10-727 Olsztyn, Poland; stompin@mp.pl; 4 Department of Pharmacodynamics, Medical University of Bialystok, 15-089 Bialystok, Poland; dariusz.pawlak@umb.edu.pl; 5 Department of Emergency Medicine, School of Medicine, Collegium Medicum, University of Warmia and Mazury in Olsztyn, 10-727 Olsztyn, Poland; rakesh.jalali@uwm.edu.pl (R.J.); dariusz.onichimowski@uwm.edu.pl (D.O.); 6 Clinical Emergency Department, Regional Specialist Hospital in Olsztyn, 10-045 Olsztyn, Poland; 7 Clinical Department of Anaesthesiology and Intensive Care, Regional Specialist Hospital in Olsztyn, 10-045 Olsztyn, Poland; 8 Branch in Ełk, University of Warmia and Mazury in Olsztyn, 10-727 Olsztyn, Poland; grazyna.piwko@uwm.edu.pl; 9 Scanmed Cardiology Center in Ełk, 19-300 Ełk, Poland; artur.zalewski@scanmed.pl; 10 National Medical Institute of the Ministry of Interior and Administration, 02-507 Warsaw, Poland; jacek.bil@cmkp.edu.pl

**Keywords:** atherosclerosis, renal artery disease, invasive angiography, long-term outcomes

## Abstract

**Background:** We aimed to characterize the population of consecutive patients undergoing coronary angiography with simultaneous renal artery angiography and assess prognostic factors at a 10 year follow-up. **Methods:** The KORONEF study was a prospective, single-center, observational, and descriptive study with 492 patients included. We analyzed several baseline demographics, clinical and periprocedural characteristics, and laboratory data, and we assessed the results of coronary angiography and renal artery angiography. **Results:** The study population consisted of 37.2% women, and the mean age was 64.4 ± 9.9 years (min. 30 years, max. 89 years). Angiography revealed significant renal artery stenosis (RAS) in 35 (7.1%) patients. Among patients with significant RAS (≥50%), we observed more women (57.1% vs. 35.7%, *p* = 0.011), and patients were older (69.1 ± 10.4 years vs. 64.0 ± 9.7 years, *p* = 0.005). In the whole population, all-cause death was reported in 29.9% of patients, myocardial infarction (MI) rate—in 11.8%, and stroke—in 4.9%. In the multivariable analysis, independent predictors of death were age 65–75 years (HR 2.88), age > 75 years (HR 8.07), diabetes (HR 1.59), previous MI (HR 1.64), chronic kidney disease (HR 2.22), unstable angina (HR 0.37), and left ventricular ejection fraction > 60% (HR 0.43). **Conclusions:** Over a 10 year follow-up, the all-cause death rate was 29.9%, showing no statistically significant differences between patients with and without significant RAS.

## 1. Introduction

The atherosclerotic process can impact multiple vascular beds within medium and large arteries, potentially narrowing the renal artery lumen. This condition, known as renal artery stenosis (RAS), can subsequently result in secondary hypertension [1,2]. In patients with coronary artery disease (CAD), the prevalence of atherosclerotic RAS is estimated to range between 11% and 23% [3]. In an autopsy study by Kuroda et al., 12.1% of individuals with ischemic stroke exhibited atherosclerotic RAS [4]. Another autopsy study conducted by Sawicki et al. reported that 8.3% of individuals with diabetes had atherosclerotic RAS [5]. Due to the concomitant atherosclerotic disease in coronary and cerebral vascular beds, individuals with atherosclerotic RAS are at heightened risk of cardiovascular events, as noted in the study by Kalra et al. [6]. Patients with atherosclerotic RAS demonstrated a significantly higher risk of CAD, heart failure, cerebrovascular events, and chronic kidney disease (CKD).

According to the findings from Salehi et al., a prospective study involving 500 participants who underwent simultaneous renal angiography following coronary angiography suggested that factors such as age over 62 years, systolic blood pressure exceeding 150 mmHg, pulse pressure surpassing 60 mmHg, and involvement of the right coronary artery may serve as independent predictors of significant RAS [7]. This justifies performing the screening renal artery angiography after coronary angiography in such patients. The RAS prevalence with any degree of stenosis in the study population was 26.2%, and significant RAS was present in 14% of patients. Dzielińska et al. analyzed a total of 333 consecutive hypertensive patients with CAD who underwent clinically indicated non-emergent coronary angiography followed by renal angiography [8]. Significant RAS (>50% lumen narrowing) was identified in 40 patients (12%), while non-significant RAS (<50%) was found in 45 (13.5%) subjects. This underscores the prevalence of RAS in many patients undergoing cardiac catheterization.

Our study aimed to assess the incidence of RAS in patients undergoing coronary angiography and analyze 10 year outcomes in this population.

## 2. Materials and Methods

### 2.1. Study Design and Participants

**Figure 1:**
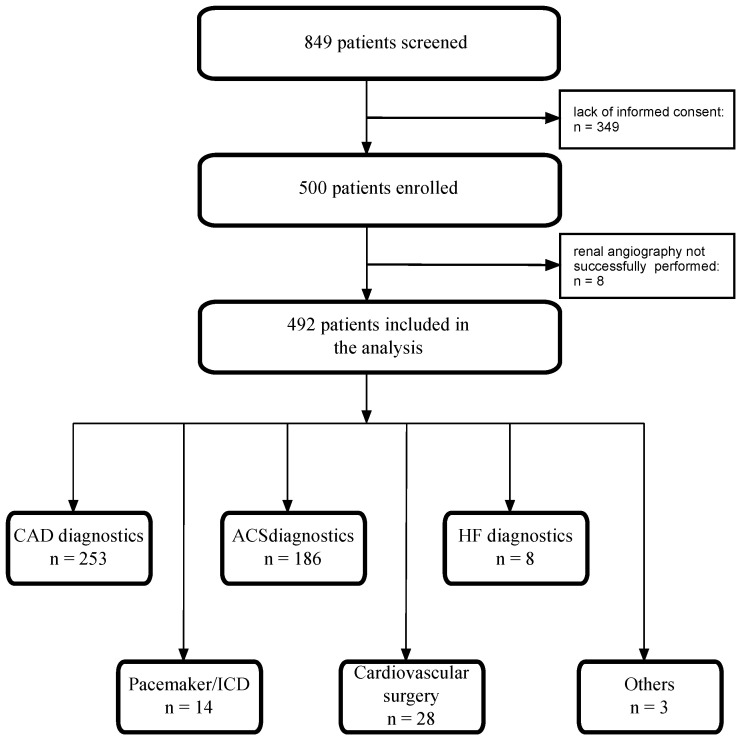
Study flowchart. CAD—coronary artery disease; ACS—acute coronary syndrome; HF—heart failure; ICD—implantable cardioverter-defibrillator.

The KORONEF study was a prospective, single-center, observational, and descriptive study. Between June and December 2011, 500 patients were consecutively enrolled, and 492 patients were included in the analysis since renal angiography was not successfully performed in 8 patients (clinical conditions: *n* = 6; renal artery anomaly: *n* = 2) (Figure 1).

Patients hospitalized due to clinical manifestations of CAD, including chronic coronary artery syndrome (CCS), as well as acute coronary artery syndrome (ACS) such as unstable angina (UA), ST-elevation myocardial infarction (STEMI), and non-ST-elevation myocardial infarction (NSTEMI), alongside patients with chronic heart failure (HF) and those qualified for other procedures (e.g., cardioverter-defibrillator implantation, cardiovascular surgeries for heart valves defects, aortic aneurysm), were included. The exclusion criterion was a lack of informed consent.

Upon admission to the cardiology department, all patients had medical history taken with particular emphasis on CAD risk factors and underwent physical examination. This was followed by blood sampling and echocardiography. Included patients underwent coronary angiography with simultaneous renal artery angiography. Depending on the patient’s clinical condition and CAD advancement, patients were qualified for conservative treatment, percutaneous coronary angioplasty (PCI), or coronary artery bypass grafting (CABG). 

### 2.2. Data Collection

We acquired information on the following comorbidities from hospital records: arterial hypertension, diabetes, dyslipidemia, previous myocardial infarction (MI), prior PCI, CKD (estimated glomerular filtration rate (eGFR) < 60 mL/min/1.73 m^2^), history of CABG, peripheral artery disease, previous stroke, smoking, and chronic obstructive pulmonary disease. We also analyzed laboratory results obtained at admission: complete blood count with differential (WBC—white blood cells, RBC—red blood cells, Hgb—hemoglobin, PLT—platelets), glucose, thyroid-stimulating hormone (TSH), N-terminal prohormone of brain natriuretic peptide (NT-proBNP), high-sensitivity C-reactive protein (hs-CRP), uric acid, troponin T, kinase creatinine (CK), CK-MB, lipid profile, creatinine, and eGFR. Finally, we summarized medications prescribed at discharge.

Echocardiographic parameters (left ventricular ejection fraction (LVEF), left ventricular end-diastolic diameter, intraventricular septal diameter, right ventricular systolic pressure, tricuspid annular plane systolic excursion) were measured with a commercially available diagnostic ultrasound device. Experienced cardiologists measured the values according to the European Association of Cardiovascular Imaging guidelines [9].

Follow-up data were collected via telephone interviews, and in missing cases, data on deaths were obtained from the Central Statistical Office (GUS).

### 2.3. Procedure Characteristics

Patients underwent coronary angiography and simultaneous angiography of renal arteries. The procedure involved puncturing either the right or left femoral artery or, in some cases, the radial artery. Following the puncture, a vascular sheath was inserted to advance the catheters under the control of an X-ray safely. Diagnostic angiographic catheters with a diameter of 6F were used, featuring appropriate curvatures adapted for intubating coronary arteries, predominantly the Judkins type (JL4, JR4) and less frequently the Amplatz type (AL1, AR1). The coronary anatomy was assessed, focusing on the presence and location of the stenosis, occurrence of total occlusion, and degree of narrowing of the arterial lumen. 

Given that coronary angiography was performed through femoral artery puncture in over 90% of patients, additional angiography of renal arteries served as a straightforward complement to the examination. Approximately 10–20 mL of contrast medium was administered to the right and left renal arteries, obtaining an angiographic image to visualize any stenoses along their course.

### 2.4. Study Endpoints

The primary endpoint of our study was the occurrence of all-cause death at 10 years. Secondary endpoints included MI, stroke, PCI, and CABG rates at 10 years.

### 2.5. Statistical Methods

Descriptive statistics were used to characterize the collected data. The normality of the distribution of continuous variables was tested using the Shapiro–Wilk test. Descriptive statistics included the mean and standard deviation for variables with a normal distribution and the median, first quartile, and third quartile for variables with a non-normal distribution. Categorical variables were presented as counts and percentages of occurrences for non-empty observations, with the percentage of non-blank observations for each parameter assessed always being at least 95%. Differences in the distribution of continuous variables between the two groups were tested using Student’s *t*-test or the Mann–Whitney test for variables with normal and non-normal distributions, respectively. Differences in the distribution of categorical variables between groups were tested using Fisher’s exact test. Survival analysis was assessed using Kaplan–Meier curves with follow-up time calculated as the time from the date of first hospitalization to the last follow-up or time to death. Cox regression analysis was used to identify predictors of death. Results were presented as hazard ratios (HR) and 95% confidence intervals for univariable analysis. Multivariable Cox regression analysis was then conducted. In the preliminary model based on univariable analysis, variables were selected with a significance level of 0.05. The multivariable Cox model was chosen using backward stepwise selection with a significance level of 0.1. Results are presented as hazard ratios (HR) and 95% confidence intervals for HR [10]. Statistical analysis was performed using the R statistical package version 3.1.2 (R Core Team, 2014). In all analyses, the significance level was set at *p* < 0.05.

## 3. Results

### 3.1. Baseline Characteristics

Between June and December 2011, 492 patients who met the inclusion criteria and did not meet the exclusion criteria were included in the study. Women constituted 37.2% (*n* = 183) of the study population, the mean age of the whole study population was 64.4 ± 9.9 years (range 30–89 years), and the mean body mass index (BMI) value was 27.9 ± 4.3 kg/m^2^. The following comorbidities were most frequent: arterial hypertension was present in 367 patients (74.6%), dyslipidemia—in 226 (45.9%), diabetes—in 128 (26.0%), previous MI—in 157 (31.9%), and CKD—in 48 (9.8%). Angiographically significant RAS (at least 50% of the lumen diameter) was observed in 35 (7.1%) patients. Patients with angiographically significant RAS were mainly females (57.1% vs. 35.7% *p* = 0.011). This population was older (69.1 ± 10.4 years vs. 64.0 ± 9.7 years, *p* = 0.005), and several comorbidities were more frequent: diabetes (40% vs. 24.9%, *p* = 0.049), previous stroke (22.9% vs. 5.3%, *p* < 0.001), CKD (28.6% vs. 8.3%, *p* < 0.001), and CKD treated with hemodialysis (5.7% vs. 0.4%, *p* = 0.027) (Table 1).

The left ventricular ejection fraction in the whole population was 52.5 ± 11.5%, and no significant differences between RAS < 50% and RAS ≥ 50% were reported between subgroups (Table 1). Patients with RAS ≥ 50% had statistically significantly lower values of hemoglobin (*p* = 0.014) and eGFR (*p* < 0.001), as well as having higher values of serum creatinine (*p* = 0.003) and hs-CRP (*p* = 0.04) (Table 2).

### 3.2. Periprocedural and Discharge Characteristics

The planned CAD diagnostics was the most common indication for coronary angiography (*n* = 253, 51.4%). Coronary angiography performed in 128 (26%) patients did not reveal any angiographically significant lesions in coronary arteries, while in 86 (23.4%) patients, the presence of three-vessel CAD was detected, including involvement of the left main stem (LMS). Additionally, 211 (46.1%) patients were qualified for percutaneous revascularization. Most often, the lesions were in the left anterior descending artery (LAD) or its collaterals (*n* = 90, 37.8%). The acute coronary syndrome was the main indication for coronarography in the RAS ≥ 50% subgroup (48.6% vs. 36.9%, *p* = 0.016). Also, in this group, two-vessel disease (40.0% vs. 27.1%, *p* = 0.009), three-vessel disease (22.9% vs. 12.3%, *p* = 0.009), and LMS stenosis (8.6% vs. 4.2% *p* = 0.009) were more frequent. These patients were also significantly more likely to undergo PCI (57.6% vs. 45.2%, *p* = 0.023) and CABG (24.2% vs. 19.3%, *p* = 0.023) (Table 3). No ad hoc renal artery revascularization was performed. After a comprehensive analysis, three patients underwent renal artery stenting.

Patients received treatment in accordance with standards, with 90.2% of patients receiving acetylsalicylic acid at discharge, 91.1%—an angiotensin-converting enzyme (ACE) inhibitor/angiotensin receptor antagonist, 92.5%—a beta-blocker, and 93.5%—a statin. Patients with RAS ≥ 50% were more likely to receive a Ca-blocker (40.0% vs. 24.1%, *p* = 0.036), a loop diuretic (37.1% vs. 16.8%, *p* = 0.003), and a vitamin K antagonist (17.1% vs. 6.6%, *p* = 0.034) at discharge, while a beta-blocker use was less frequent (82.9% vs. 93.2%, *p* = 0.038) (Table 4).

### 3.3. Ten-Year Follow-Up Data

The median follow-up period was 10.2 years (min. 5.9 years; max. 10.3 years). In the whole population, all-cause death was observed in 29.9% of patients, MI—in 11.8%, and stroke—in 4.9%. No statistically significant differences were found between the groups (RAS < 50% vs. ≥50%) (Table 5). No adverse events with implanted renal stents were reported.

**Figure 2:**
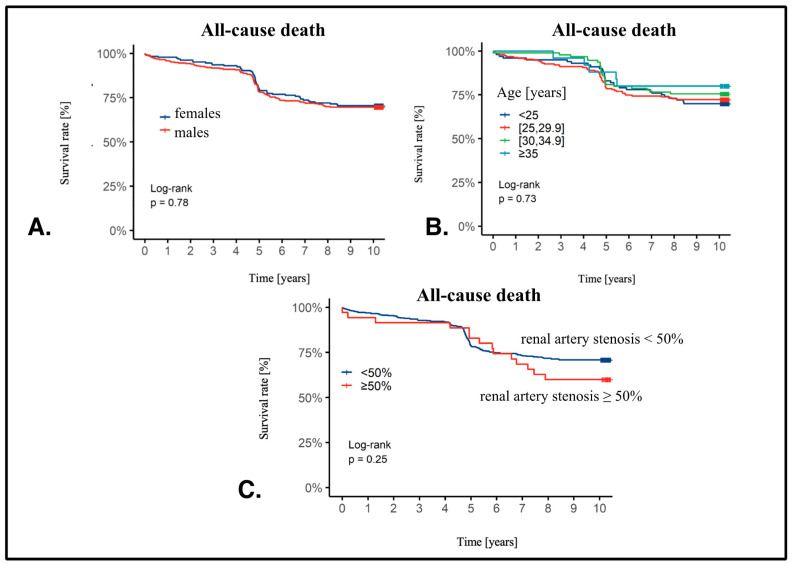
(**A**). Kaplan–Meier curve showing survival depending on the sex. K—female, M—male. (**B**) Kaplan–Meier curve showing survival depending on the BMI value. (**C**) Kaplan-Meier curve showing survival depending on the RAS stenosis.

**Figure 3:**
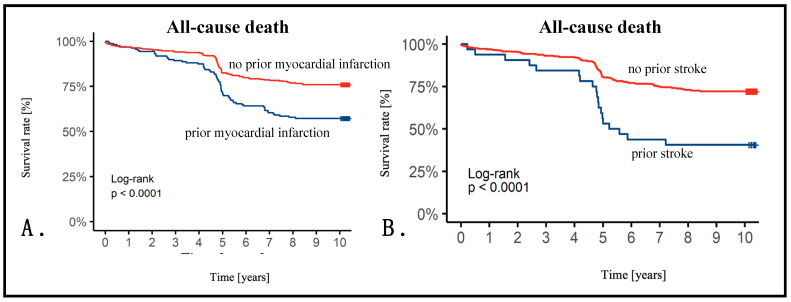
(**A**). Kaplan–Meier curve showing survival depending on the prior myocardial infarction history. (**B**) Kaplan–Meier curve showing survival depending on the prior stroke history.

We presented Kaplan–Meier curves with survival rates in additional subgroups. There was no difference in females vs. males (males: HR 1.05, 95%CI 0.75–1.46, *p* = 0.78). Also, BMI did not influence survival (BMI 25–29.9: HR 0.95, 95%CI 0.61–1.49; BMI 30–34.9: HR 0.81, 95%CI 0.47–1.39; BMI ≥ 35: HR 0.65, 95%CI 0.25–1.68, *p* = 0.73) or RAS presence (RAS ≥ 50%: HR 1.38, 95% CI 0.79–2.39, *p* = 0.25) (Figure 2). However, we observed a significant impact on survival in patients with prior MI (prior MI: HR 2.00, 95% CI 1.45–2.56, *p* < 0.001) or stroke (prior stroke: HR 2.70, 95% CI 1.67–4.35, *p* < 0.001) (Figure 3).

### 3.4. Cox Analysis

Finally, we analyzed predictive factors for all-cause death at 10 years. The multivariable analysis results are depicted in Table 6 (univariable analyses are presented in Tables S1–S3).

In the whole population, in the multivariable model, statistically significant predictors included age 65–75 years (HR 2.88), age > 75 years (HR 8.07), diabetes (HR 1.59), previous MI (HR 1.64), CKD (HR 2.22), UA (HR 0.37), and LV ejection fraction > 60% (HR 0.43) (Table 6). In patients with RAS ≥ 50%, in the multivariable model, only dyslipidemia was found to be a statistically significant predictor of all-cause death (HR 5.52) (Table S4).

## 4. Discussion

In our study, the angiographically significant RAS (at least 50% of the lumen diameter) was observed in 35 (7.1%) patients. The median follow-up period was 10.2 years (min. 5.9 years; max. 10.3 years). In the whole population, all-cause death was observed in 29.9% of patients, MI—in 11.8%, and stroke—in 4.9%. No statistically significant differences were found between the groups (RAS <50% vs. ≥ 50%). No adverse events associated with implanted renal stents were reported.

In the KORONEF study, the population undergoing coronary angiography consisted of 37.2% women and 62.8% men, with a mean age of 64.4 ± 9.9 years (min. 30 years, max. 89 years). This is a typical population undergoing invasive diagnostics as part of CAD diagnostics, which has also been observed in other studies [11]. The study population was characterized by multi-morbidity. Arterial hypertension was present in 367 patients (74.6%), dyslipidemia—in 226 (45.9%), diabetes—in 128 (26.0%), previous MI—in 157 (31.9%), and CKD—in 48 (9.8%). These are well-known CAD risk factors and/or progression factors [12,13]. Similar data can be found in the literature, as in the EURECA study [14,15,16]. Despite such numerous risk factors, no significant atherosclerotic changes were found in 26% of patients. This indicates the appropriate qualification of patients for coronary angiography.

In the KORONEF study, 23.4% of patients had three-vessel CAD, including LMS involvement, 37.6% had two-vessel disease, and 38.1% had one-vessel disease. The study enrolled patients in 2011, which is probably why as many as 19.7% of patients underwent CABG and 46.1% PCI. In coronary angiography, lesions were most often located in the LAD or its branches (*n* = 90, 37.8%), with bare metal stents used more often in patients undergoing PCI (145 BMS vs. 86 DES). The only P2Y12 receptor inhibitor used was clopidogrel, not ticagrelor or prasugrel, which are currently recommended in patients with ACS [17]. This could have impacted the prognosis as the new P2Y12 inhibitors tend to improve outcomes. Moreover, as other studies show, using radial access in patients with comorbidities is safe and feasible [11]. The median follow-up period was 10.2 years (min. 5.9 years; max. 10.3 years). In the entire population, death from any cause was observed in 29.9% of patients, MI—in 11.8%, and stroke—in 4.9%. As previously mentioned, 36.9% were patients with ACS. In the KORONEF study, patients had a similar prognosis as patients with MINOCA and STEMI in the 9 year follow-up study by Buller et al. (17.9% vs. 24.1%, HR 1.15, 95% CI 0.67–1.94, *p* = 0.61) [18]. Other studies presented similar results [19,20]. Goy et al. presented 10 year results of the randomized SIMA study comparing PCI with BMS vs. CABG (LIMA) as a treatment option for proximal LAD stenosis. Death rates were 8.1% vs. 6.8% (*p* = 0.4), and the percentage of MI—4.8% vs. 5.1% (*p* = 0.9). These results are definitely lower than our population’s; however, the study population was very small (62 and 59 patients) [21]. Slightly higher values were presented by Muller et al., showing 5 year results of a study in which, in patients with proximal LAD stenosis, the decision about PCI vs. CABG was made based on the FFR result. In the group treated conservatively, the percentage of deaths was 5.3%, MI—0.4%, and repeated revascularization—2.0%. In turn, in the group treated invasively, the percentage of deaths was 9.6%, MI—1.2%, and repeated revascularizations—15.9% [22]. Similar results (7 years) were presented by Yamashita et al. The percentage of cardiovascular events ranged from 27% to 33% [23]. In the KORONEF study, older patients had a statistically significantly worse prognosis (age > 65 years: HR 3.35, 95% CI 1.69–6.61, *p* < 0.0001); age > 75 years: HR 8.05, 95% CI 4.09–15.9, *p* < 0.0001), as well as patients with a history of MI (patients without a history of MI: HR 0.50, 95% CI 0.36–0.69, *p* < 0.0001) and stroke (patients with no history of stroke: HR 0.37, 95% CI 0.23–0.60, *p* < 0.0001). 

The KORONEF study tackled an interesting topic, namely, the issue of multiple vascular beds involvement. Atherosclerosis is a major cause of global mortality, responsible for nearly 10 million deaths each year. The plaques mainly develop in the coronary and carotid arteries, the aorta, and the peripheral arteries of the lower limbs [24,25]. The first reports on the coexistence of CAD and peripheral artery disease come from the 1980s [26]. This topic is of utmost interest. In the REACH study, patients with CAD and ≥1 other vascular bed had a higher incidence of cardiovascular events than CAD patients alone [27]. The SMART study showed that cardiovascular calcifications on cardiac valves and in multiple vascular beds in the chest and abdomen were linked with a higher incidence of cardiovascular events [28]. This was also confirmed in other studies [29]. 

The KORONEF study assessed the incidence of stenoses in the renal arteries in patients undergoing CAD diagnostics. In the analyzed population of 492 patients, angiographically significant stenosis in the renal arteries was confirmed in 7.1% of cases. Among patients with significant RAS, there were more women (57.1% vs. 35.7%, *p* = 0.011), and patients were older (69.1 ± 10.4 years vs. 64.0 ± 9.7 years, *p* = 0.005). This might be a bit surprising since one expected more dominant atherosclerosis in men. In patients with RAS ≥ 50%, coronary angiography was performed more often due to ACS (48.6% vs. 36.9%, *p* = 0.016) and two-vessel disease (40.0% vs. 27.1%, *p* = 0.009). These patients also underwent PCI more often (57.6% vs. 45.2%, *p* = 0.023). Here, the advanced renal artery disease was strictly linked with CAD advancement. However, there were no statistically significant differences in prognosis between the groups. This may be partly due to the small number of patients with RAS (*n* = 35).

Renal artery disease (RAD) is generally considered when RAS is >50–60%, although additional functional assessment based on hemodynamic criteria is indicated [30]. The incidence of RAD increases with age and is mainly related to atherosclerosis. It is associated with male sex, hypertension, smoking, diabetes, and CKD, as well as atherosclerosis of the aorta, iliac arteries, and coronary arteries [31,32]. It may occur in 5–10% of the general population, although it is more common in high-risk populations [33]. Approximately 20% of patients have bilateral disease, or it may affect the only functioning kidney [30]. In the Cardiovascular Health Study, RAS > 60% was detected using Doppler ultrasonography in 9.1% of men and 5.5% of women [34]. Of 450 patients who underwent cardiac catheterization for suspected CAD, the incidence of RAD was higher in people with CAD compared to patients without CAD (9.9% vs. 4.1%). It correlated with the number of affected coronary arteries [35]. This rate is comparable to the rate observed in the KORONEF study. Nevertheless, some differences in the incidence of RAS may be mainly attributed to the different screening criteria used in the studies. Some studies have used ≥70% as the RAS diagnostic cut-off point, and current guidelines propose a value of ≥60% [30,36,37]. In the KORONEF study, we adopted ≥ 50% as an important stenosis criterion for early detection of RAS [38,39,40], considering that RAD of atherosclerotic etiology is a progressive disease, even if it is not angiographically significant. The diagnosis of RAS provides influential information on patient management [41]. The large REN-ACS study also accepted a value of ≥50% [42]. As already mentioned, RAD of atherosclerotic etiology is progressive, and the risk of progression is the greatest in the case of high-grade stenosis and the co-occurrence of severe hypertension and diabetes [43]. Less than 10% of patients with RAS develop significant stenosis or occlusion within 5 years [44], and deterioration of renal function is rare in the case of unilateral RAS, more common in the case of bilateral RAS or one functional kidney (after 2 years, respectively, 3%, 18%, and 55%) [45]. Life expectancy is shorter in RAD patients, even without CKD, because they most often die from an acute cardiovascular event [33,46]. Patients who progress to CKD have much higher mortality rates [30,47].

In most studies, the authors focused, as shown, on identifying predicting factors of the presence of significant RAS. The KORONEF study identified risk factors of an unfavorable prognosis in this population during long-term follow-up, i.e., 10 years. Within the population of patients with RAS < 50% in the multivariable Cox regression model, statistically significant predictors of death from any cause included age 65–75 years (HR 2.21), age > 75 years (HR 5.99), diabetes (HR 1.56), CKD (HR 1.75), and left ventricular ejection fraction >60% (HR 0.34). Conversely, in the population of patients with RAS ≥ 50% in the multivariable Cox regression model, only dyslipidemia emerged as a statistically significant predictor of death from any cause (HR 5.52). An interesting paper by Allison et al. was published regarding lipid profile and atherosclerosis in multiple vascular beds. They identified in computed tomography the prevalence of calcium in various locations as follows: 31.6% in the carotids, 58.7% in the coronaries, 40.5% in the thoracic aorta, 55.0% in the abdominal aorta, 16.8% in the iliac arteries, 57.0% in the renal arteries, 8.3% in the mitral annulus, and 23.6% in the aortic annulus. In men, a 1 S.D. increase in the total to HDL cholesterol ratio was significantly linked to 36% higher odds of having any calcium in the abdominal aorta and 29% higher odds in the iliac arteries. In women, the same increase was significantly associated with odds ratios of 1.66 for the presence of any calcium in the thoracic aorta and 1.41 in the abdominal aorta [48].

Unfortunately, few studies have addressed the topic of risk factors in RAS patients, primarily focusing on patients undergoing revascularization of renal arteries. Dregoesc et al., in a 10 year follow-up, demonstrated that patients who underwent renal artery stenting had a total mortality rate of 44.6% [49]. Independent predicting factors for this outcome included age (OR 1.1; 95% CI 1.0–1.2; *p* = 0.01), male sex (OR 7.9; 95% CI 1.9–43.5; *p* = 0.008), post-stenting CKD class 3b-5 (OR 5.8; 95% CI 1.5–27.9; *p* = 0.01), and post-revascularization uncontrolled hypertension (OR 8.9; 95% CI 1.7–63.5; *p* = 0.01), irrespective of the presence of diabetes and CAD. In an analogous group, Zachrisson et al. reported a mortality rate of 60% with a median follow-up of 11 years and 7 months [50]. The KORONEF study further confirmed the significantly worse prognosis in patients with CKD, necessitating close monitoring.

### Study Limitations

The study had several limitations. The most significant were the small population of patients with RAS and the lack of assessment of the functional significance of stenosis in the renal arteries. Another factor that contributed to the lack of a homogeneous group was the inclusion of not only patients with CAD but all those undergoing coronary angiography. A benefit of this approach was the evaluation of the prevalence of RAS, not only in patients with advanced CAD. Additionally, a small percentage of patients underwent renal artery revascularization. Nevertheless, this paradoxically reflects the current state of knowledge, where the indications for renal artery revascularization are very limited. Finally, it is worth noting that the use of only clopidogrel, instead of newer P2Y12 receptor inhibitors, may have been associated with a worse prognosis.

## 5. Conclusions

Patients undergoing coronary angiography were typically males over 60 years old with multiple comorbidities, including hypertension, dyslipidemia, diabetes, previous MI, and obesity. Despite receiving optimal medical pharmacotherapy, they experienced a relatively high rate of adverse events such as MI, stroke, and death. In the KORONEF study, 7.1% of patients had angiographically significant RAS (≥50%). Among those with significant stenosis, females were more common, and patients were older, with a higher likelihood of diabetes, a history of stroke, and CKD, including those treated by hemodialysis. This subgroup frequently underwent coronary angiography due to ACS, with a predominance of two-vessel disease and a higher likelihood of PCI.

Over a 10 year follow-up, the overall mortality rate was 29.9%, showing no statistically significant difference between patients with and without significant RAS. In the whole study population, predictors of death included age 65–75 years, age over 75 years, diabetes, CKD, and left ventricular ejection fraction over 60%. However, dyslipidemia was the only statistically significant predicting factor in patients with significant RAS.

## Figures and Tables

**Table 1:** Baseline characteristics.

Parameter	Study Population N = 492 (%)	Patients with RAS < 50% N = 457 (%)	Patients with RAS ≥ 50% N = 35 (%)	*p*
Females	183 (37.2%)	163 (35.7%)	20 (57.1%)	0.011
Age (years)	64.4 ± 9.9	64.0 ± 9.7	69.1 ± 10.4	0.005
BMI (kg/m^2^)	27.9 ± 4.3	28.0 ± 4.3	26.9 ± 3.2	0.307
Arterial hypertension	367 (74.6%)	339 (74.2%)	28 (80.0%)	0.446
Dyslipidemia	226 (45.9%)	212 (46.4%)	14 (40.0%)	0.465
Diabetes	128 (26.0%)	114 (24.9%)	14 (40.0%)	0.049
Obesity	147 (29.9%)	135 (29.5%)	12 (34.3%)	0.554
Previous MI	157 (31.9%)	144 (31.5%)	13 (37.1%)	0.491
Previous stroke	32 (6.5%)	24 (5.3%)	8 (22.9%)	<0.001
Peripheral artery disease	25 (5.1%)	23 (5.0%)	2 (5.7%)	0.696
Chronic kidney disease	48 (9.8%)	38 (8.3%)	10 (28.6%)	<0.001
Chronic kidney disease treated with hemodialysis	4 (0.8%)	2 (0.4%)	2 (5.7%)	0.027
Previous CABG	21 (4.3%)	17 (3.7%)	4 (11.4%)	0.054
Previous PCI	105 (21.3%)	96 (21.0%)	9 (25.7%)	0.512
**Echocardiography results**				
LV ejection fraction (%)	52.5 ± 11.5	52.7 ± 11.5	51.3 ± 11,9	0.475
LVEDd [mm]	49 [46–54]	49 [46–54.5]	48.5 [42.2–51.8]	0.135
IVSd [mm]	10 [8–12]	10 [8–12.7]	10 [8–12]	0.868
RVSP [mmHg]	39 [32.8–45.8]	39 [35–46]	26 [22–30]	0.202
TAPSE [mm]	17.5 [13.2–21.0]	18 [14–21]	17 [13–19]	0.381

Results presented as mean ± standard deviation or median [interquartile range]; RAS—renal artery stenosis; BMI—body mass index; MI—myocardial infarction; CABG—coronary artery bypass grafting; PCI—percutaneous coronary intervention; LV—left ventricular; LVEDd—left ventricular end-diastolic diameter; IVSd—intraventricular septal diameter; RVSP—right ventricular systolic pressure; TAPSE—tricuspid annular plane systolic excursion.

**Table 2:** Biochemical tests.

Parameter	Study Population N = 492 (%)	Patients with RAS < 50% N = 457 (%)	Patients with RAS ≥ 50% N = 35 (%)	*p*
Erythrocytes [10^12^/L]	4.7 [4.3–5.0]	4.7 [4.3–5.0]	4.4 [4.2–4.8]	0.026
Hemoglobin [g/dL]	14 [13–14.9]	14 [13–14.9]	13 [12.6–14.5]	0.014
Glucose [mg/dL]	103 [93–126]	103 [93–126]	113 [93.5–139]	0.316
Creatinine [mg/dL]	0.9 [0.8–1.0]	0.9 [0.8–1.0]	1.1 [0.8–1.4]	0.003
eGFR [mL/min]	81 [67–93]	82 [68–93]	65 [52.2–79.2]	<0.001
K^+^ [mmol/L]	4.3 [4.1–4.7]	4.3 [4.1–4.7]	4.3 [4.1–4.6]	0.711
Na^+^ [mmol/L]	141 [139–143]	141 [139–143]	140 [138–142]	0.298
Total cholesterol [mg/dL]	178 [149.8–211]	177 [149–210.8]	179.5 [153.2–224]	0.508
LDL [mg/dL]	101.5 [76–135.2]	101 [76–135]	111 [77.5–141]	0.573
HDL [mg/dL]	51 [42–62]	51 [42–62]	46 [40–57.5]	0.186
Triglycerides [mg/dL]	116 [86–161]	115 [85.8–161]	124 [88.5–158.5]	0.704
TSH [μIU/mL]	1.3 [0.8–2.1]	1.3 [0.8–2.1]	1.0 [0.6–1.5]	0.276
NT-proBNP [pg/mL]	1307 [627–4334]	1148 [638–4295]	1699 [934–8368]	0.605
hs-CRP [mg/L]	0.2 [0.1–0.5]	0.2 [0.1–0.5]	0.4 [0.2–0.8]	0.040
Uric acid [mg/dL]	8.4 [8.3–8.6]	8.4 [8.3–8.6]	-	-
Max. TnT [μg/L]	0.7 [0.1–1.4]	0.6 [0.1–1.1]	1.0 [0.2–1.6]	0.320
Max. CK-MB [IU/mL]	23 [16.2–42]	23 [17–42]	13 [10–24]	0.171

Results presented as mean ± standard deviation or median [interquartile range]; RAS—renal artery stenosis; HDL—high-density lipoprotein; LDL—low-density lipoprotein; TSH—thyroid-stimulating hormone; NT-proBNP—N-terminal prohormone of brain natriuretic peptide; hsCRP—high sensitivity C-reactive protein.

**Table 3:** Periprocedural data.

Parameter	Study Population N = 492 (%)	Patients with RAS < 50% N = 457 (%)	Patients with RAS ≥ 50% N = 35 (%)	*p*
Coronary angiography indications				
Planned coronary artery disease diagnostics	253 (51.4%)	238 (52.1%)	15 (42.9%)	0.016
Acute coronary syndrome	186 (37.8%)	169 (36.9%)	17 (48.6%)	
Heart failure diagnostics	8 (1.6%)	7 (1.5%)	1 (2.9%)	
Pacemaker/ICD qualification	14 (2.8%)	14 (3.1%)	0	
Cardiovascular surgery (heart valve defect, ascending aorta aneurysm)	28 (5.7%)	26 (5.7%)	2 (5.7%)	
Others	3 (0.6%)	3 (0.7%)	0	
Coronary angiography results				
No significant atherosclerotic lesions *	128 (26.0%)	122 (26.7%)	6 (17.1%)	0.009
One-vessel disease	140 (38.1%)	136 (29.8%)	4 (11.4%)	
Two-vessel disease	138 (37.6%)	124 (27.1%)	14 (40.0%)	
Three-vessel disease	64 (17.4%)	56 (12.3%)	8 (22.9%)	
Left main stem	22 (6.0%)	19 (4.2%)	3 (8.6%)	
Qualification for revascularization				
Pharmacological treatment	191 (38.8%)	8 (24.2%)	8 (24.2%)	0.023
PCI	211 (46.1%)	19 (57.6%)	19 (57.6%)	
CABG	90 (19.7%)	8 (24.2%)	8 (24.2%)	
Location of lesions treated by PCI (*n* = 238)				
Left main stem	1 (0.4%)	1 (0.5%)	0	0.628
Left anterior descending artery/ diagonal branches	90 (37.8%)	80 (41.7%)	10 (52.6%)	
Left circumflex artery/marginal branches	59 (24.8%)	55 (28.6%)	4 (21.1%)	
Intermediate artery	4 (1.7%)	4 (2.1%)	0	
Right coronary artery	82 (34.5%)	75 (39.1%)	7 (36.8%)	
Venous graft	2 (0.8%)	2 (1.0%)	0	
Coronary bifurcation	3 (0.6%)	3 (1.6%)	0	0.65
Number of implanted bare metal stents				
1	120 (82.8%)	109 (81.9%)	11 (91.7%)	0.758
2	21 (14.5%)	20 (15.1%)	1 (8.3%)	
3	4 (2.8%)	4 (3.0%)	0	
Number of implanted drug-eluting stents				
1	77 (89.5%)	70 (90.9%)	7 (77.8%)	0.153
2	8 (9.3%)	6 (7.8%)	2 (22.2%)	
3	1 (1.2%)	1 (1.3%)	0	
TIMI after PCI (*n* = 211)		N = 192	N = 19	
0	11 (5.2%)	11 (5.7%)	0	0.723
1	3 (1.4%)	3 (1.6%)	0	
2	1 (0.5%)	1 (0.5%)	0	
3	196 (92.9%)	177 (92.2%)	19 (100%)	
Periprocedural complications (PCI) (*n* = 211)		N = 192	N = 19	
No reflow/slow reflow	9 (4.3%)	9 (4.7%)	0	0.998
Stent thrombosis	1 (0.5%)	1 (0.5%)	0	1

* no atherosclerotic lesions or lesions < 40%, TIMI—Thrombolysis In Myocardial Infarction; RAS—renal artery stenosis; PCI—percutaneous coronary intervention, CABG—coronary artery bypass grafting, ICD—implantable cardioverter-defibrillator.

**Table 4:** Medications at discharge.

Parameter	Study Population N = 492 (%)	Patients with RAS < 50% N = 457 (%)	Patients with RAS ≥ 50% N = 35 (%)	*p*
Acetylsalicylic acid	444 (90.2%)	410 (89.7%)	34 (97.1%)	0.999
Clopidogrel	304 (61.8%)	280 (61.3%)	24 (68.6%)	0.091
ACE inhibitor	427 (86.8%)	399 (87.3%)	28 (80.0%)	0.170
Angiotensin antagonist	21 (4.3%)	20 (4.4%)	1 (2.9%)	0.290
Beta-blocker	455 (92.5%)	426 (93.2%)	29 (82.9%)	0.999
Ca-blocker	124 (25.2%)	110 (24.1%)	14 (40.0%)	0.090
Statin	460 (93.5%)	429 (93.9%)	31 (88.6%)	0.476
Fibrate	17 (3.5%)	17 (3.7%)	0	0.999
Loop diuretic	90 (18.3%)	77 (16.8%)	13 (37.1%)	<0.001
Thiazide	51 (10.4%)	49 (10.7%)	2 (5.7%)	0.713
Mineralocorticoid receptor antagonist	66 (13.4%)	62 (13.6%)	4 (11.4%)	0.785
Vitamin K antagonist	36 (7.3%)	30 (6.6%)	6 (17.1%)	0.006
Oral hypoglycemic drugs	87 (17.7%)	79 (17.3%)	8 (22.9%)	0.384
Insulin	45 (9.1%)	40 (8.8%)	5 (14.3%)	0.579

ACE—angiotensin-converting enzyme.

**Table 5:** The outcomes in 10 years follow-up.

Endpoint	Study Population N = 492 (%)	Patients with RAS < 50% N = 457 (%)	Patients with RAS ≥ 50% N = 35 (%)	*p*
Death	147 (29.9%)	133 (29.2%)	14 (40.0%)	0.177
MI	58 (11.8%)	54 (11.8%)	4 (11.4%)	>0.999
Stroke	24 (4.9%)	21 (4.6%)	3 (8.6%)	0.24
CABG	37 (4.9%)	36 (7.9%)	1 (2.9%)	0.502
PCI	82 (16.7%)	77 (16.9%)	5 (14.3%)	0.691

RAS—renal artery stenosis; MI—myocardial infarction; CABG—coronary artery bypass grafting; PCI—percutaneous coronary intervention.

**Table 6:** Factors predicting death in the 10 year follow-up—multivariable Cox analysis.

	Study Population N = 492 (%)		
Parameter	HR	95% CI	*p*-Value
**Age:**			
30–55 years	—	—	—
55–60 years	2.12	0.92, 4.86	0.076
60–65 years	1.24	0.48, 3.16	0.7
65–75 years	2.88	1.31, 6.34	0.008
75–90 years	8.07	3.65, 17.8	<0.001
**Diabetes**	1.59	1.05–2.42	0.028
**Previous myocardial infarction**	1.64	1.09–2.44	0.017
**Chronic kidney disease**	2.22	1.33–3.70	0.002
**Coronary angiography indications:**			
CAD	—	—	—
NSTEMI	0.93	0.53, 1.61	0.8
STEMI	1.73	0.99, 3.02	0.052
Unstable angina	0.37	0.15, 0.93	0.034
**Left ventricular ejection fraction**			
≤40	—	—	—
[40, 50]	0.53	0.32, 0.87	0.011
[50, 60]	0.51	0.29, 0.90	0.020
>60	0.43	0.23, 0.78	0.006
**LDL cholesterol**			
≤100	—	—	—
[100, 129]	0.61	0.35, 1.06	0.080
[129, 159]	0.77	0.46, 1.29	0.3
[159, 465]	0.96	0.51, 1.81	>0.9

HR = hazard ratio, CI = confidence interval, CAD—coronary artery disease; STEMI—ST-elevation myocardial infarction; NSTEMI—non-ST-elevation myocardial infarction.

## Data Availability

Data are available from the corresponding author on request.

## Supplementary Materials

The following supporting information can be downloaded at https://www.mdpi.com/article/10.3390/jcm13123374/s1. Table S1: Univariable Cox regression for death in the whole population. Table S2: Univariable Cox regression for death in patients with RAS < 50%. Table S3: Univariable Cox regression for death in patients with RAS ≥ 50%. Table S4: Multivariable Cox regression for death in patients with RAS < 50% and RAS ≥ 50%.
