# Supplementary material for: Ten-Year Outcomes in Patients Undergoing Simultaneous Coronary and Renal Angiography—Does Renal Artery Stenosis Matter?
Source: J Clin Med. 2024 Jun 7;13(12):3374. doi: 10.3390/jcm13123374 (PMC11204998; doi:10.3390/jcm13123374)
Supplement: Supplementary file 1 [file jcm-13-03374-s001.zip › jcm-2988591-supplementary.pdf]

Table S1: Univariable Cox regression for death in the whole population;

	Univariable analysis			Multivariable analysis		
**Characteristic**	**HR[1]**	**95% CI[1]**	**p-value**	**HR[1]**	**95% CI[1]**	**p-value**
**Sex**						
Female	—	—				
Male	1.05	0.75, 1.46	0.8			
**Age**						
(30,55]	—	—		—	—	
(55,60]	1.77	0.85, 3.70	0.13	2.12	0.92, 4.86	0.076
(60,65]	1.30	0.58, 2.89	0.5	1.24	0.48, 3.16	0.7
(65,75]	3.35	1.69, 6.61	<0.001	2.88	1.31, 6.34	0.008
(75,90]	8.05	4.09, 15.9	<0.001	8.07	3.65, 17.8	<0.001
**BMI**						
<25	—	—				
[25,29.9]	0.95	0.61, 1.49	0.8			
[30,34.9]	0.81	0.47, 1.39	0.4			
≥35	0.65	0.25, 1.68	0.4			
**Diabetes**						
yes	—	—		—	—	
no	0.49	0.35, 0.68	<0.001	0.63	0.42, 0.95	0.028
**HTN**						
yes	—	—				
no	0.86	0.58, 1.26	0.4			
**Nicotine**						
yes	—	—				
no	1.44	0.98, 2.11	0.063			
**Obesity**						
yes	—	—				
no	0.98	0.69, 1.40	>0.9			
**Dyslipidemia**						
yes	—	—				
no	1.49	1.07, 2.08	0.018			
**Prior MI**						
yes	—	—		—	—	
no	0.50	0.36, 0.69	<0.001	0.61	0.41, 0.92	0.017
**Prior stroke**						
yes	—	—				
no	0.37	0.23, 0.60	<0.001			
**PAD**						
yes	—	—				
no	0.57	0.31, 1.02	0.059			
**Dialysis**						
yes	—	—				
no	3,319,756	0.00, Inf	>0.9			
**CKD**						
yes	—	—		—	—	
no	0.37	0.25, 0.56	<0.001	0.45	0.27, 0.75	0.002
**prior CABG**						
yes	—	—				
no	0.57	0.30, 1.07	0.082			

Table S1: Univariable Cox regression for death in the whole population;

	Univariable analysis			Multivariable analysis		
**Characteristic**	**HR[1]**	**95% CI[1]**	**p-value**	**HR[1]**	**95% CI[1]**	**p-value**
**prior PCI**						
yes	—	—				
no	0.79	0.55, 1.15	0.2			
**STEMI**						
yes	—	—				
no	0.90	0.59, 1.39	0.6			
**NSTEMI**						
yes	—	—				
no	0.79	0.51, 1.24	0.3			
**UA**						
yes	—	—				
no	0.89	0.52, 1.52	0.7			
**SCA**						
yes	—	—				
no	0.30	0.12, 0.74	0.009			
**AF**						
yes	—	—		—	—	
no	0.37	0.23, 0.58	<0.001	0.49	0.28, 0.85	0.011
**Coronary angiography**						
3-VD	—	—				
LM	1.23	0.57, 2.67	0.6			
**BMS number**						
0	—	—				
1	0.98	0.67, 1.43	>0.9			
2	1.71	0.89, 3.26	0.11			
3	1.87	0.46, 7.59	0.4			
**DES number**						
0	—	—				
1	0.62	0.37, 1.03	0.067			
2	0.72	0.18, 2.91	0.6			
3	0.00	0.00, Inf	>0.9			
**POBA**						
0	—	—				
1	0.86	0.55, 1.33	0.5			
2	2.71	0.38, 19.4	0.3			
**TIMI after pci**						
0	—	—				
1	0.43	0.05, 3.56	0.4			
2	0.00	0.00, Inf	>0.9			
3	0.40	0.17, 0.93	0.033			
**Indications**						
CAD	—	—		—	—	
NSTEMI	1.05	0.65, 1.69	0.8	0.93	0.53, 1.61	0.8
STEMI	1.22	0.78, 1.92	0.4	1.73	0.99, 3.02	0.052
UA	0.39	0.16, 0.96	0.040	0.37	0.15, 0.93	0.034
**RAS**						

Table S1: Univariable Cox regression for death in the whole population;

	Univariable analysis			Multivariable analysis		
**Characteristic**	**HR[1]**	**95% CI[1]**	**p-value**	**HR[1]**	**95% CI[1]**	**p-value**
<50%	—	—				
≥50%	1.38	0.79, 2.39	0.3			
**Echo EF**						
≤40	—	—		—	—	
(40,50]	0.53	0.34, 0.83	0.005	0.53	0.32, 0.87	0.011
(50,60]	0.44	0.26, 0.72	0.001	0.51	0.29, 0.90	0.020
>60	0.28	0.16, 0.47	<0.001	0.43	0.23, 0.78	0.006
**hsCRP**						
≤0.1	—	—				
(0.1,0.2]	1.02	0.60, 1.73	>0.9			
(0.2,0.5]	1.07	0.66, 1.74	0.8			
(0.5,82]	1.50	0.93, 2.40	0.10			
**LDL chol**						
≤100	—	—		—	—	
(100,129]	0.55	0.35, 0.88	0.013	0.61	0.35, 1.06	0.080
(129,159]	0.70	0.44, 1.11	0.13	0.77	0.46, 1.29	0.3
(159,465]	0.80	0.48, 1.35	0.4	0.96	0.51, 1.81	>0.9
**Glu**						
≤80	—	—				
(80,100]	0.95	0.34, 2.63	>0.9			
(100,140]	1.33	0.48, 3.67	0.6			
(140,200]	1.37	0.47, 4.03	0.6			
>200	2.74	0.89, 8.41	0.078			
**eGFR**						
≤60	—	—				
>60	0.30	0.21, 0.42	<0.001			

[1]HR = Hazard Ratio, CI = Confidence Interval

Table S2: Univariable Cox regression for death in patients with RAS < 50%;

	Univariable analysis			Multivariable analysis		
**Characteristic**	**HR[1]**	**95% CI[1]**	**p-value**	**HR[1]**	**95% CI[1]**	**p-value**
**Sex**						
Female	—	—				
Male	1.02	0.72, 1.46	>0.9			
**Age**						
(30,55]	—	—		—	—	
(55,60]	1.64	0.78, 3.44	0.2	1.77	0.82, 3.79	0.14
(60,65]	1.08	0.47, 2.46	0.9	0.80	0.32, 1.98	0.6
(65,75]	2.89	1.44, 5.77	0.003	2.21	1.07, 4.58	0.032
(75,90]	7.69	3.88, 15.3	<0.001	5.99	2.88, 12.5	<0.001
**BMI**						
<25	—	—				
[25,29.9]	0.96	0.60, 1.54	0.9			
[30,34.9]	0.83	0.47, 1.46	0.5			
≥35	0.66	0.26, 1.72	0.4			
**Diabetes**						
yes	—	—		—	—	
no	0.51	0.36, 0.73	<0.001	0.64	0.42, 0.97	0.037
**HTN**						
yes	—	—				
no	0.81	0.54, 1.21	0.3			
**Nicotine**						
yes	—	—				
no	1.42	0.95, 2.13	0.084			
**Obesity**						
yes	—	—				
no	1.02	0.70, 1.48	>0.9			
**Dyslipidemia**						
yes	—	—				
no	1.36	0.96, 1.93	0.082			
**Prior MI**						
yes	—	—				
no	0.54	0.38, 0.76	<0.001			
**Prior stroke**						
yes	—	—				
no	0.37	0.21, 0.64	<0.001			
**PAD**						
yes	—	—				
no	0.63	0.33, 1.20	0.2			
**Dialysis**						
yes	—	—				
no	3,296,188	0.00, Inf	>0.9			
**CKD**						
yes	—	—		—	—	
no	0.34	0.21, 0.53	<0.001	0.57	0.33, 0.98	0.041
**Prior CABG**						
yes	—	—				

Table S2: Univariable Cox regression for death in patients with RAS < 50%;

	Univariable analysis			Multivariable analysis		
**Characteristic**	**HR[1]**	**95% CI[1]**	**p-value**	**HR[1]**	**95% CI[1]**	**p-value**
no	0.49	0.25, 0.97	0.041			
**Prior PCI**						
yes	—	—				
no	0.81	0.54, 1.20	0.3			
**STEMI**						
yes	—	—				
no	0.89	0.57, 1.39	0.6			
**NSTEMI**						
yes	—	—				
no	0.78	0.48, 1.28	0.3			
**UA**						
yes	—	—				
no	0.94	0.53, 1.67	0.8			
**SCA**						
yes	—	—				
no	0.37	0.14, 1.00	0.051			
**AF**						
yes	—	—		—	—	
no	0.34	0.21, 0.56	<0.001	0.46	0.26, 0.80	0.007
**Coronary angiography**						
3-VD	—	—				
LM	1.48	0.64, 3.42	0.4			
**BMS number**						
0	—	—				
1	0.95	0.63, 1.43	0.8			
2	1.58	0.80, 3.14	0.2			
3	1.91	0.47, 7.76	0.4			
**DES number**						
0	—	—				
1	0.66	0.39, 1.14	0.13			
2	0.49	0.07, 3.52	0.5			
3	0.00	0.00, Inf	>0.9			
**POBA**						
0	—	—				
1	0.87	0.55, 1.37	0.5			
2	2.85	0.40, 20.4	0.3			
**TIMI after PCI**						
0	—	—				
1	0.42	0.05, 3.52	0.4			
2	0.00	0.00, Inf	>0.9			
3	0.39	0.17, 0.90	0.027			
**Indications**						
CAD	—	—				
NSTEMI	1.10	0.67, 1.82	0.7			

Table S2: Univariable Cox regression for death in patients with RAS < 50%;

	Univariable analysis			Multivariable analysis		
**Characteristic**	**HR[1]**	**95% CI[1]**	**p-value**	**HR[1]**	**95% CI[1]**	**p-value**
STEMI	1.29	0.80, 2.06	0.3			
UA	0.37	0.13, 1.00	0.051			
**Echo EF**						
≤40	—	—		—	—	
(40,50]	0.50	0.31, 0.82	0.005	0.42	0.26, 0.70	<0.001
(50,60]	0.47	0.28, 0.80	0.005	0.49	0.28, 0.86	0.012
>60	0.28	0.16, 0.49	<0.001	0.34	0.19, 0.61	<0.001
**hsCRP**						
≤0.1	—	—				
(0.1,0.2]	1.12	0.64, 1.94	0.7			
(0.2,0.5]	1.11	0.67, 1.85	0.7			
(0.5,82]	1.61	0.97, 2.66	0.066			
**LDL chol**						
≤100	—	—		—	—	
(100,129]	0.50	0.30, 0.84	0.009	0.61	0.35, 1.06	0.079
(129,159]	0.70	0.42, 1.14	0.15	0.79	0.47, 1.32	0.4
(159,465]	0.85	0.50, 1.46	0.6	0.92	0.48, 1.75	0.8
**Glu**						
≤80	—	—				
(80,100]	1.14	0.36, 3.68	0.8			
(100,140]	1.67	0.52, 5.35	0.4			
(140,200]	1.41	0.40, 4.90	0.6			
>200	3.41	0.97, 12.0	0.056			
**eGFR**						
≤60	—	—				
>60	0.30	0.20, 0.43	<0.001			

[1]HR = Hazard Ratio, CI = Confidence Interval

Table S3: Univariable Cox regression for death in patients with RAS ≥ 50%.

	UNivariable analysis			Multivariable analysis		
**Characteristic**	**HR[1]**	**95% CI[1]**	**p-value**	**HR[1]**	**95% CI[1]**	**p-value**
**Sex**						
Female	—	—				
Male	1.56	0.55, 4.46	0.4			
**Age**						
(30,55]	—	—				
(55,60]	1.00	0.00, Inf	>0.9			
(60,65]	446,050,386	0.00, Inf	>0.9			
(65,75]	311,122,673	0.00, Inf	>0.9			
(75,90]	302,429,703	0.00, Inf	>0.9			
**BMI**						
<25	—	—				
[25,29.9]	0.60	0.13, 2.68	0.5			
[30,34.9]	0.43	0.05, 4.18	0.5			
≥35						
**Diabetes**						
yes	—	—				
no	0.43	0.15, 1.25	0.12			
**HTN**						
yes	—	—				
no	2.02	0.63, 6.47	0.2			
**Nicotine**						
yes	—	—				
no	4.02	0.53, 30.8	0.2			
**Obesity**						
yes	—	—				
no	0.87	0.29, 2.60	0.8			
**Dyslipidemia**						
yes	—	—		—	—	
no	5.85	1.30, 26.3	0.021	5.52	1.22, 25.0	0.027
**Prior MI**						
yes	—	—				
no	0.34	0.12, 0.98	0.046			
**Prior stroke**						
yes	—	—				
no	0.40	0.13, 1.20	0.10			
**PAD**						
yes	—	—				
no	0.26	0.06, 1.18	0.081			
**Dialysis**						
yes	—	—				
no	74,103,887	0.00, Inf	>0.9			
**CKD**						
yes	—	—				
no	0.68	0.23, 2.02	0.5			

Table S3: Univariable Cox regression for death in patients with RAS ≥ 50%.

	UNivariable analysis			Multivariable analysis		
**Characteristic**	**HR[1]**	**95% CI[1]**	**p-value**	**HR[1]**	**95% CI[1]**	**p-value**
**Prior CABG**						
yes	—	—				
no	1.67	0.22, 12.8	0.6			
**Prior PCI**						
yes	—	—				
no	0.83	0.26, 2.64	0.7			
**STEMI**						
yes	—	—				
no	0.51	0.11, 2.26	0.4			
**NSTEMI**						
yes	—	—				
no	0.99	0.31, 3.17	>0.9			
**UA**						
yes	—	—				
no	0.48	0.11, 2.14	0.3			
**SCA**						
yes	—	—				
no	0.00	0.00, Inf	>0.9			
**AF**						
yes	—	—				
no	0.50	0.16, 1.61	0.2			
**Coronary angiography**						
3-VD	—	—				
LM	0.47	0.06, 4.08	0.5			
**BMS number**						
0	—	—		—	—	
1	1.48	0.48, 4.53	0.5	1.48	0.48, 4.53	0.5
2	18.9	1.65, 217	0.018	12.3	1.07, 143	0.044
3						
**DES number**						
0	—	—				
1	0.27	0.04, 2.08	0.2			
2	1.18	0.15, 9.08	0.9			
3						
**POBA**						
0	—	—				
1	0.95	0.21, 4.25	>0.9			
2						
**Indications**						
CAD	—	—				
NSTEMI	0.65	0.14, 3.00	0.6			
STEMI	1.09	0.23, 5.04	>0.9			
UA	0.44	0.06, 3.48	0.4			
**Echo EF**						

Table S3: Univariable Cox regression for death in patients with RAS ≥ 50%.

	UNivariable analysis			Multivariable analysis		
Characteristic	HR[1]	95% CI[1]	p-value	HR[1]	95% CI[1]	p-value
≤40	—	—				
(40,50]	0.90	0.23, 3.50	0.9			
(50,60]	0.29	0.05, 1.73	0.2			
>60	0.16	0.02, 1.56	0.11			
**hsCRP**						
≤0.1	—	—				
(0.1,0.2]	0.19	0.02, 2.09	0.2			
(0.2,0.5]	0.86	0.16, 4.72	0.9			
(0.5,82]	0.72	0.14, 3.58	0.7			
**LDL chol**						
≤100	—	—				
(100,129]	0.63	0.17, 2.40	0.5			
(129,159]	0.46	0.10, 2.18	0.3			
(159,465]	0.45	0.06, 3.57	0.4			
**Glu**						
≤80	—	—				
(80,100]	0.28	0.03, 2.59	0.3			
(100,140]	0.26	0.03, 2.29	0.2			
(140,200]	0.56	0.06, 5.17	0.6			
>200						
**eGFR**						
≤60	—	—				
>60	0.33	0.11, 0.96	0.043			

[1]HR = Hazard Ratio, CI = Confidence Interval

Supplementary Table S4. Multivariable analysis in RAS subgroups.

	Study population N = 492 (%)			Patients with RAS < 50% N = 457 (%)			Patients with RAS ≥ 50% N = 35 (%)		
Parameter	HR	95% CI	p-value	HR	95% CI	p-value	HR	95% CI	p-value
**Age**									
[30,55]	—	—	—	—	—	—	—	—	—
[55,60]	2.12	0.92, 4.86	0.076	1.77	0.82, 3.79	0.14	—	—	—
[60,65]	1.24	0.48, 3.16	0.7	0.80	0.32, 1.98	0.6	—	—	—
[65,75]	2.88	1.31, 6.34	0.008	2.21	1.07, 4.58	0.032	—	—	—
[75,90]	8.07	3.65, 17.8	<0.001	5.99	2.88, 12.5	<0.001	—	—	—
**Diabetes**									
yes	—	—	—	—	—	—	—	—	—
no	0.63	0.42, 0.95	0.028	0.64	0.42, 0.97	0.037	—	—	—
**Dyslipidemia**									
yes	—	—	—	—	—	—	5.52	1.22, 25.0	0.027
no	—	—	—	—	—	—	—	—	—
**Previous myocardial infarction**									
yes	—	—	—	—	—	—	—	—	—
no	0.61	0.41, 0.92	0.017	—	—	—	—	—	—
**Chronic kidney disease**									
yes	—	—	—	—	—	—	—	—	—

no	0.45	0.27, 0.75	0.002	0.57	0.33, 0.98	0.041	—	—	—
**Bare metal stents (number)**									
0	—	—	—	—	—	—	—	—	—
1	—	—	—	—	—	—	1.48	0.48, 4.53	0.5
2	—	—	—	—	—	—	12.3	1.07, 143	0.044
**Coronagraphy indications**									
CAD	—	—	—	—	—	—	—	—	—
NSTEMI	0.93	0.53, 1.61	0.8	—	—	—	—	—	—
STEMI	1.73	0.99, 3.02	0.052	—	—	—	—	—	—
UA	0.37	0.15, 0.93	0.034	—	—	—	—	—	—
**Left ventricular ejection fraction**									
≤40	—	—	—	—	—	—	—	—	—
[40,50]	0.53	0.32, 0.87	0.011	0.42	0.26, 0.70	<0.001	—	—	—
[50,60]	0.51	0.29, 0.90	0.020	0.49	0.28, 0.86	0.012	—	—	—
>60	0.43	0.23, 0.78	0.006	0.34	0.19, 0.61	<0.001	—	—	—
**LDL cholesterol**									
≤100	—	—	—	—	—	—	—	—	—
[100,129]	0.61	0.35, 1.06	0.080	0.61	0.35, 1.06	0.079	—	—	—
[129,159]	0.77	0.46, 1.29	0.3	0.79	0.47, 1.32	0.4	—	—	—
[159,465]	0.96	0.51, 1.81	>0.9	0.92	0.48, 1.75	0.8	—	—	—
